# Microcirculatory Response to Changes in Venoarterial Extracorporeal Membrane Oxygenation Pump Flow: A Prospective Observational Study

**DOI:** 10.3389/fmed.2021.649263

**Published:** 2021-04-07

**Authors:** Tzu-Jung Wei, Chih-Hsien Wang, Wing-Sum Chan, Chi-Hsiang Huang, Chien-Heng Lai, Ming-Jiuh Wang, Yih-Sharng Chen, Can Ince, Tzu-Yu Lin, Yu-Chang Yeh

**Affiliations:** ^1^Department of Anesthesiology, National Taiwan University Hospital, College of Medicine, National Taiwan University, Taipei, Taiwan; ^2^Department of Surgery, National Taiwan University Hospital, College of Medicine, National Taiwan University, Taipei, Taiwan; ^3^Department of Anesthesiology, Far Eastern Memorial Hospital, New Taipei, Taiwan; ^4^Department of Intensive Care, Erasmus MC University Medical Center Rotterdam, Rotterdam, Netherlands

**Keywords:** microcirculation, extracorporeal membrane oxygenation, hemodynamics, cardiogenic shock, critical care

## Abstract

**Background:** Venoarterial extracorporeal membrane oxygenation (VA-ECMO) pump flow is crucial for maintaining organ perfusion in patients with cardiogenic shock, but VA-ECMO pump flow optimization remains as a clinical challenge. This study aimed to investigate the response of sublingual microcirculation to changes in VA-ECMO pump flow.

**Methods:** Sublingual microcirculation was measured before and after changing VA-ECMO pump flow according to the treatment plan of ECMO team within 24 h and at 24-48 h after VA-ECMO placement. In clinical events of increasing VA-ECMO pump flow, those events with increased perfused vessel density (PVD) were grouped into group A, and the others were grouped into group B. In clinical events of decreasing VA-ECMO pump flow, those events with increased PVD were grouped into group C, and the others were grouped into group D.

**Results:** Increased PVD was observed in 60% (95% CI, 38.5–81.5%) of the events with increasing VA-ECMO pump flow. The probability of increasing PVD after increasing VA-ECMO pump flow were higher in the events with a PVD < 15 mm/mm^2^ at baseline than those with a PVD ≥ 15 mm/mm^2^ [100% (95% CI, 54.1–100%) vs. 42.9% (95% CI, 17.7–71.1%), *P* = 0.042]. Other microcirculatory and hemodynamic parameters at baseline did not differ significantly between group A and B or between group C and D.

**Conclusion:** This study revealed contradictory and non-contradictory responses of sublingual microcirculation to changes in VA-ECMO pump flow. Tandem measurements of microcirculation before and after changing VA-ECMO pump flow may help to ensure a good microcirculation.

## Introduction

Venoarterial Extracorporeal Membrane Oxygenation (VA-ECMO) has become a promising option for bridge support in patients with acute cardiopulmonary failure (1–3). An inadequate low VA-ECMO pump flow leads to abnormal tissue perfusion and poor prognosis, and flow optimization is essential to maintaining adequate tissue and brain perfusion (4). In addition, high pump flow–related complications such as kidney injury, cerebral stroke, and hemorrhage are associated with high morbidity and mortality (5, 6).

Poor microcirculatory flow or function may indicate tissue hypoperfusion or hypoxia (7, 8). Our previous study and that conducted by Kara et al. revealed that a microcirculatory unresponsiveness to VA-ECMO predicted adverse outcomes (9, 10), and we found that mortality was higher in VA-ECMO patients with a perfused vessel density (PVD) less than 15 mm/mm^2^ (9). Akin et al. identified an association between microcirculation and successful weaning from VA-ECMO in late weaning process (11), but no study has reported the effects of changes in pump flow on microcirculation at early stage of VA-ECMO support. In current clinical practice, VA-ECMO pump flow is adjusted by achieving preset goals of macro-hemodynamic parameters like mean arterial pressure (MAP) and pulse pressure (12, 13). However, dissociation between sublingual microcirculation and macrocirculation has been observed in shock states (7, 14), and microcirculatory dysfunction can co-exist with normal macrocirculatory parameters (10). Investigating microcirculation after increasing or weaning VA-ECMO pump flow is crucial to ensure adequate tissue perfusion. This study aimed to investigate the responses of sublingual microcirculation to changes in VA-ECMO pump flow.

## Materials and Methods

### Study Design and Patient Enrollment

This prospective observational study was approved by the Research Ethics Committee of National Taiwan University Hospital (approval number: 201703011RINA, approval date: April 28, 2017) and registered on the ClinicalTrials.gov protocol registration system (ID: NCT03210818). It was conducted at a university medical center between November 2017 and December 2018, consistent with STROBE guidelines (15). Participants were selected from patients receiving ECMO support on the basis of eligibility screening conducted within 12 h following ECMO placement. Patients who received VA-ECMO support and were above 20 years old were included. Patients were excluded if they declined to participate, had received re-implantation of ECMO, died within 12 h, or had circumstance that prevented sublingual microcirculation from being measured within 24 h after initiating VA-ECMO, such as those in which placement occurred in the evening or the research nurse was on leave. Written informed consent was obtained from patients' legally authorized representatives before study enrollment.

### VA-ECMO Components and Treatment Goals

For all enrolled patients, placement and the principal components of the VA-ECMO were the same as described in our previous study (9). To avoid possible malperfusion of the distal limb, an antegrade distal perfusion catheter was used when the mean pressure of the superficial femoral artery was below 50 mm Hg (16). All patients received standard VA-ECMO management and routine intensive care unit (ICU) care. Heparin was continuously administered to maintain an activated clotting time of 160–180 s if no active bleeding or other complications were observed. The ECMO team adjusted the VA-ECMO pump flow according to their treatment goal to maintain a MAP >60 mm Hg, central venous oxygen saturation (ScvO_2_) >70%, central venous pressure <15 mm Hg, and lactate level of <3 mmol/L; and to avoid a urine output of <0.5 ml/kg/h, pulse pressure >10 mm Hg, and ECMO-induced hemolysis; or to wean the patients off VA-ECMO support.

### Record of Clinical Information

The following data were recorded: age, gender, height, body weight, sequential organ failure assessment (SOFA) score (17), indications for VA-ECMO, VA-ECMO pump flow, heart rate, MAP, ScvO_2_, lactate level, activated clotting time, hemoglobin level, fluid balance, and inotropic score. The inotropic score was calculated as 100 × epinephrine dose (mcg/kg/min) + 100 × norepinephrine dose (mcg/kg/min) + dopamine dose (mcg/kg/min) + dobutamine dose (mcg/kg/min) (18). The length of ICU and hospital stay, as well as survival status at 28 days were also recorded.

### Analysis of Microcirculation Videos and Grouping of Microcirculatory Responses

Sublingual microcirculation videos were recorded using an incident dark-field video microscope (CytoCam, Braedius Medical, Huizen, Netherlands) (19). Analysis of sublingual microcirculation videos was performed according to the international consensus guidelines for performing sublingual microcirculation by a Task Force of the European Society for Intensive Care Medicine (20). At each time point, five video sequences (length: 6 s each) were recorded at different sublingual sites and were digitally stored with code numbers to ensure the anonymity of patient information. Subsequent offline analyses were performed by a single observer blinded to patient information. Two or three sequences with appropriate image quality were selected for analysis using the semi-automated analysis software package Automated Vascular Analysis 3.0 (21). In accordance with the afore mentioned consensus guidelines (20), the following parameters were investigated: (a) total vessel density (TVD; vessels <20 μm), (b) PVD, and (c) proportion of perfused vessels (PPV). The software was used to automatically calculate TVD. The calculation of PVD was semiautomated using the procedure described in our previous study (9, 22).

When the ECMO team decided to increase or decrease the VA-ECMO pump flow according to their treatment goal within 24 h and at 24 to 48 h after VA-ECMO placement, the sublingual microcirculation videos were recorded before changing the VA-ECMO pump flow and 5 min after the changes. In clinical events of increasing VA-ECMO pump flow, those events with increased PVD were grouped into group A, and the others were grouped into group B. In clinical events of decreasing VA-ECMO pump flow, those events with increased PVD were grouped into group C, and the others were grouped into group D.

### Statistical Analysis

All statistical analyses were performed using SPSS version 20 (IBM, Armonk, NY, USA). Categorical variables were described as number (percentage) and were compared using chi-square tests or Fisher's exact tests as appropriate. Continuous variables were expressed as medians (interquartile range) and compared using independent-samples Mann–Whitney test and the median test. The 95% confidence interval (CI) of the proportion was calculated with binomial exact calculations (23, 24). Association between two continuous variables was compared with Pearson's r correlation analysis. All the *P*-values were not adjusted in this observational pilot study. A *P*-value of < 0.05 indicated a significant difference.

## Results

### Patient Distribution and Characteristics

**Figure 1:**
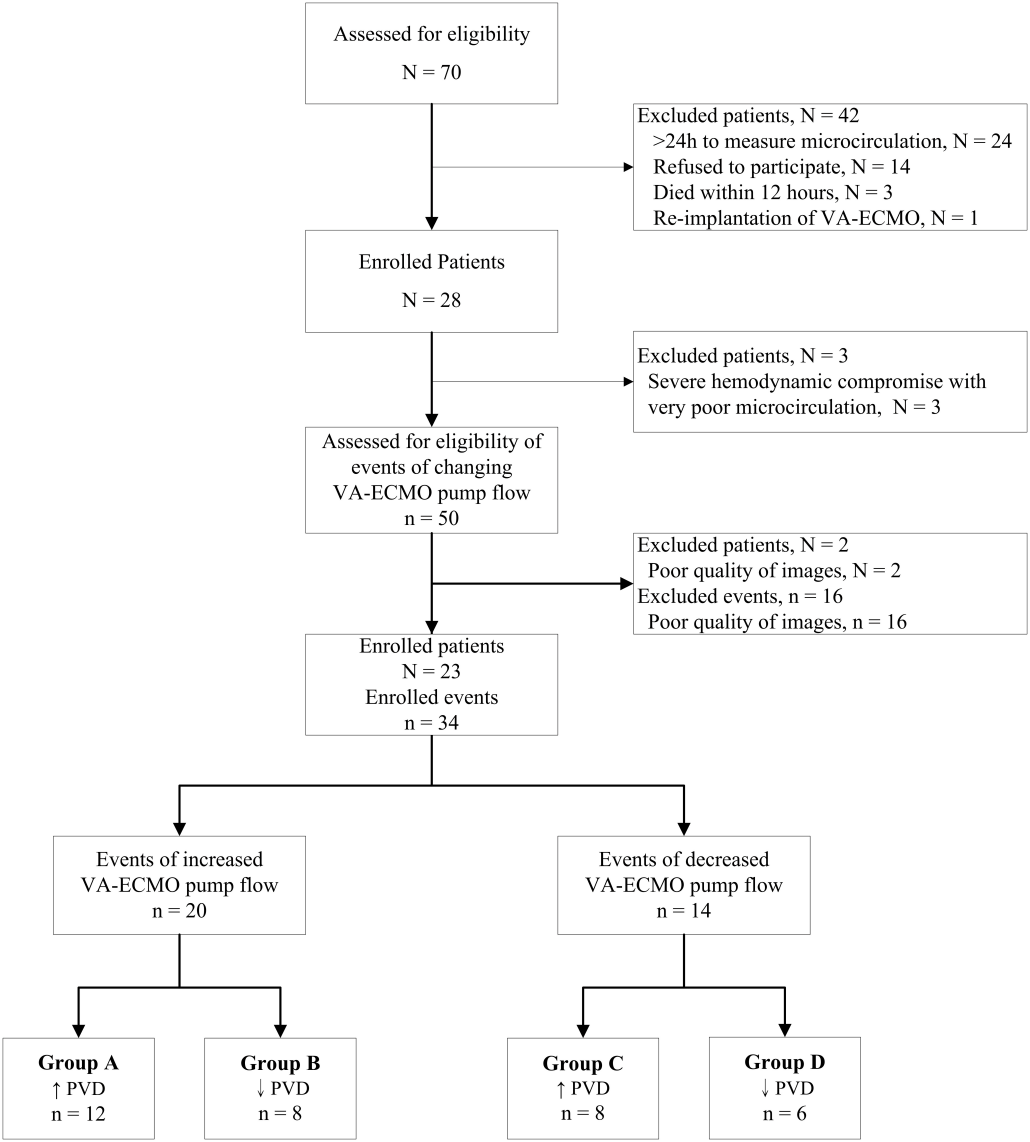
Consort flow chart of analyzed events in patients receiving venoarterial extracorporeal membrane oxygenation. *N*, number of patients; *n*, number of events of changing VA-ECMO pump flow; PVD, perfused vessel density; VA-ECMO, venoarterial extracorporeal membrane oxygenation.

A total of 70 patients receiving VA-ECMO were considered for inclusion in this trial. A total of 47 patients were excluded, and a total of 34 events with good quality of microcirculation images were analyzed (Figure 1). The patient characteristics and indications of VA-ECMO of the 23 enrolled patients are presented in Table 1. The 28-day survival rate of the enrolled patients was 52%.

**Table 1:** Patients characteristics.

**Group**	**Total**	**28-day survivors**	**28-day non-survivors**	***P*-values**
n	23	12	11	
Female/male	5/18	2/10	3/8	0.640
Age	64 (55–73)	64.5 (53–72.5)	64 (55–73)	1.000
Height	168 (160–175)	168 (160–175)	167 (160–175)	1.000
Weight	66.9 (57.9–74.7)	68 (58.3–84.7)	66.9 (56.2–74.7)	1.000
VA-ECMO flow (L/min)	2.6 (2.1–3.2)	2.3 (1.9–2.9)	2.9 (2.3–3.3)	0.220
Heart rate (bpm)	97 (79–110)	96 (78–108)	100 (90–121)	0.684
MAP (mm Hg)	76 (71–84)	75 (65–78)	79 (75–87)	0.220
Pulse pressure (mm Hg)	36 (21–55)	36 (23–62)	36 (19–45)	1.000
Hemoglobin level (g/dL)	11 (10–12)	12 (11–12)	11 (9–13)	0.214
Platelet (K/μL)	118 (68–201)	131 (89–237)	100 (32–131)	0.214
Lactate (mmol/L)	3.0 (2.0–8.0)	2.5 (2.0–8.5)	4 (3.0–8.0)	0.684
Inotropic score	14 (7–32)	9 (7–20)	20 (4–35)	0.220
SOFA score	13 (10–15)	11 (8–13)	14 (13–16)	0.027
Heart failure, n(%)	13 (57%)	7 (58%)	6 (55%)	1.000
E-CPR, n(%)	10 (43%)	5 (42%)	5 (45%)	1.000

*Data are presented as number or median (interquartile range). P-values were calculated using Fisher's exact test and independent-samples median tests. E-CPR, extracorporeal cardiopulmonary resuscitation; MAP, mean arterial pressure; SOFA, sequential organ failure assessment; VA-ECMO, venoarterial extracorporeal membrane oxygenation*.

### Microcirculatory Response to Increasing VA-ECMO Pump Flow

**Figure 2:**
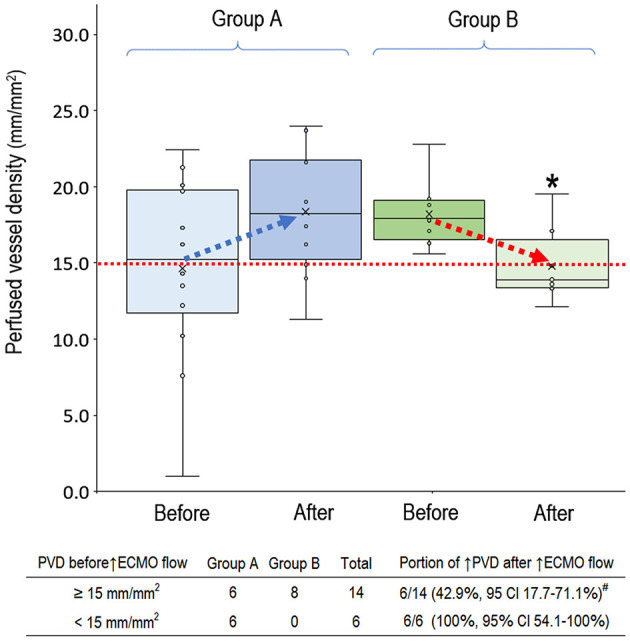
Perfused vessel density before and after increasing venoarterial extracorporeal membrane oxygenation pump flow. Group A (*n* = 12), PVD increased after increasing VA-ECMO pump flow; Group B (*n* = 8), PVD decreased after increasing VA-ECMO pump flow. PVD before increasing ECMO pump flow did not differ significantly between group A and B (*P* = 0.238). PVD was higher in the group A than in group B after increasing ECMO pump flow (*P* = 0.025). Patients with PVD < 15 mm/mm^2^ had a higher probability to increase PVD after increasing VA-ECMO pump flow (*P* = 0.042). ^*^P < 0.05 comparison between group A and B using independent-samples median tests. ^#^*P* < 0.05 comparison between PVD ≥ 15 mm/mm^2^ vs. PVD < 15 mm/mm^2^ using Fisher's exact test. CI, confidence interval; *n*, number of events; PVD, perfused vessel density; VA-ECMO, venoarterial extracorporeal membrane oxygenation.

**Figure 3:**
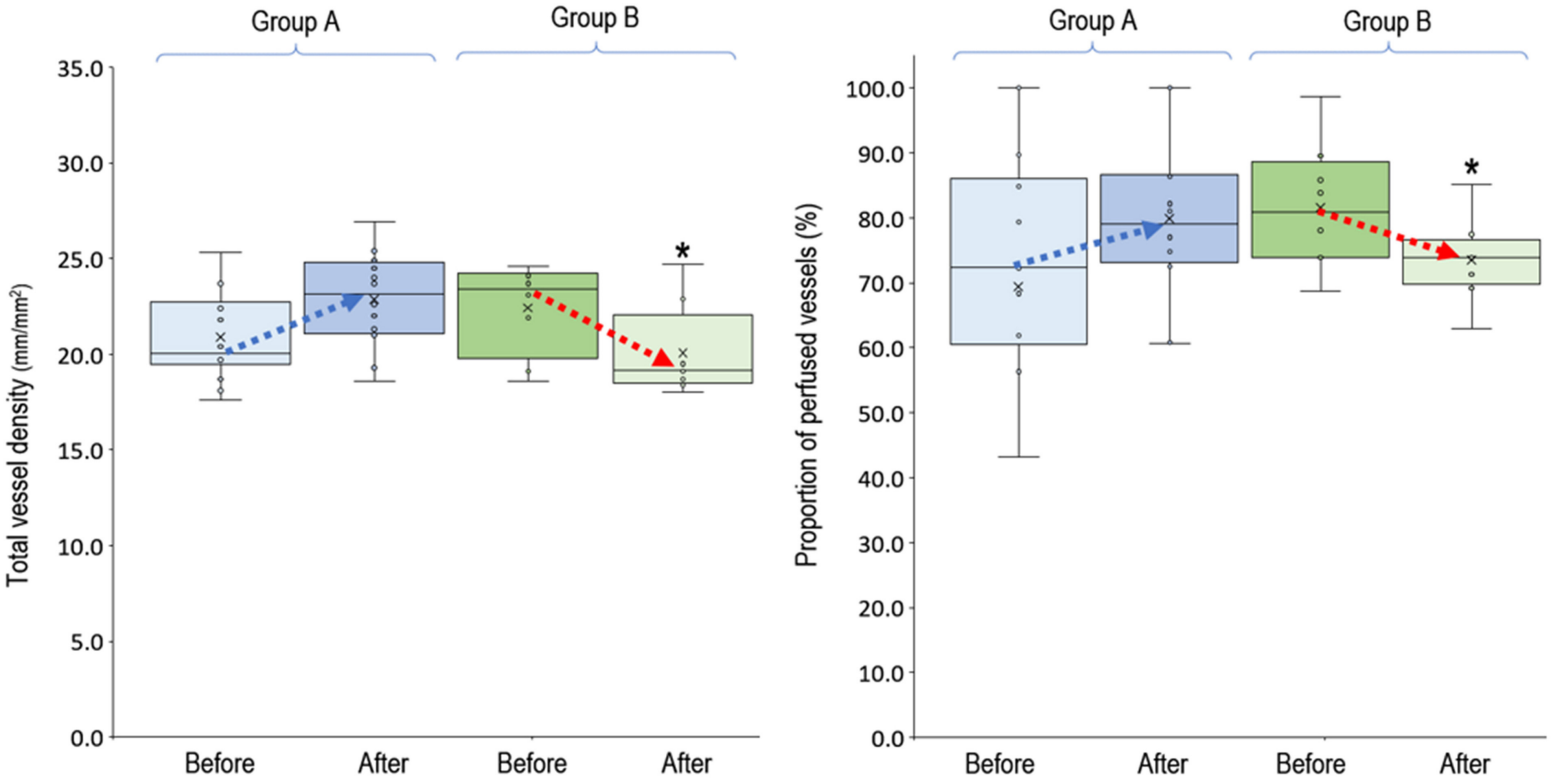
Total vessel density and proportion of perfused vessels before and after increasing venoarterial extracorporeal membrane oxygen pump flow. Group A (*n* = 12), PVD increased after increasing VA-ECMO pump flow; group B (*n* = 8), PVD decreased after increasing VA-ECMO pump flow. ^*^P < 0.05 comparison between group A and B using independent-samples median tests. *n*, number of events; PVD, perfused vessel density; PPV, proportion of perfused vessels; TVD, total vessel density; VA-ECMO, venoarterial extracorporeal membrane oxygenation.

**Figure 4:**
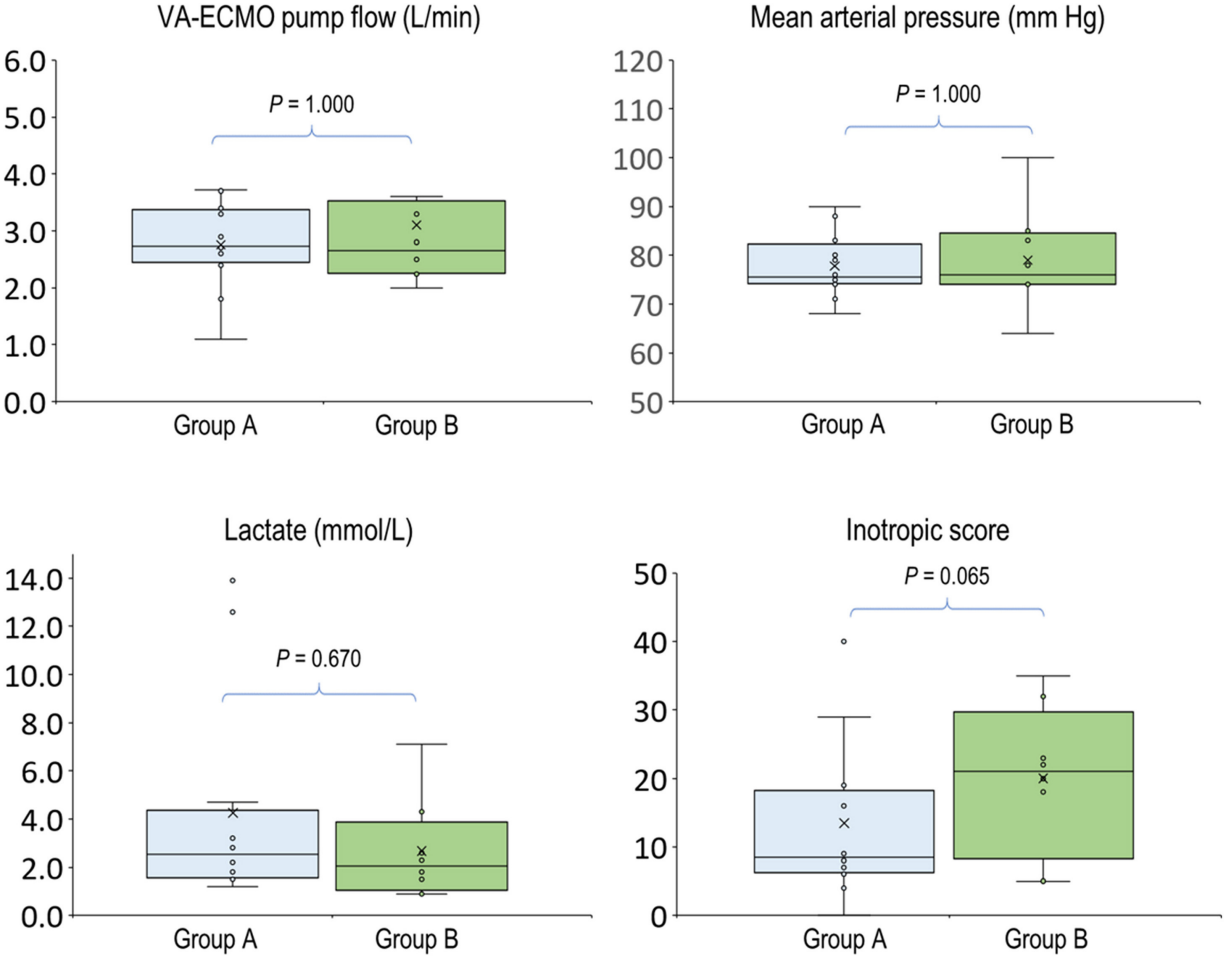
Hemodynamic parameters before increasing venoarterial extracorporeal membrane oxygenation pump flow. Group A (*n* = 12), PVD increased after increasing VA-ECMO pump flow; group B (*n* = 8), PVD decreased after increasing VA-ECMO pump flow. *P*-values were calculated using independent-samples median tests. *n*, number of events; VA-ECMO, venoarterial extracorporeal membrane oxygenation.

In the 20 clinical events of increasing VA-ECMO pump flow, we observed only 12 (60%, 95% CI, 38.5–81.5%) events with increased PVD (Figure 2). In *post-hoc* analysis, these events were divided into two groups according to their PVD before changing the VA-ECMO pump flow: PVD < 15 mm/mm^2^ and PVD ≥ 15 mm/mm^2^, respectively. The probability of increasing PVD after increasing VA-ECMO pump flow were higher in the events with PVD < 15 mm/mm^2^ at baseline than those with PVD ≥ 15 mm/mm^2^ at baseline [100% (95% CI, 54.1–100%) vs. 42.9% (95% CI, 17.7–71.1%), *P* = 0.042. The values of PVD before increasing VA-ECMO pump flow were negatively correlated to the changes of PVD after increasing VA-ECMO pump flow (Pearson correlation coefficient = −0.706, *P* = 0.001). TVD and PPV before and after increasing VA-ECMO pump flow are presented in Figure 3. The values of TVD before increasing VA-ECMO pump flow were negatively correlated to the changes of TVD after increasing VA-ECMO pump flow (Pearson correlation coefficient = −0.641, *P* = 0.002). The values of PPV before increasing VA-ECMO pump flow were negatively correlated to the changes of PPV after increasing VA-ECMO pump flow (Pearson correlation coefficient = −0.872, *P* < 0.001). Figure 4 summarizes the VA-ECMO pump flow, MAP, lactate level, and inotropic score before increasing the VA-ECOM pump flow between group A and B. All the parameters did not differ significantly between group A and B. ScvO_2_ before increasing the VA-ECOM pump flow did not differ significantly between group A and B [77 (69–82)% vs. 83 (77–93) %, *P* = 0.065).

### Microcirculatory Response to Deceasing VA-ECMO Pump Flow

**Figure 5:**
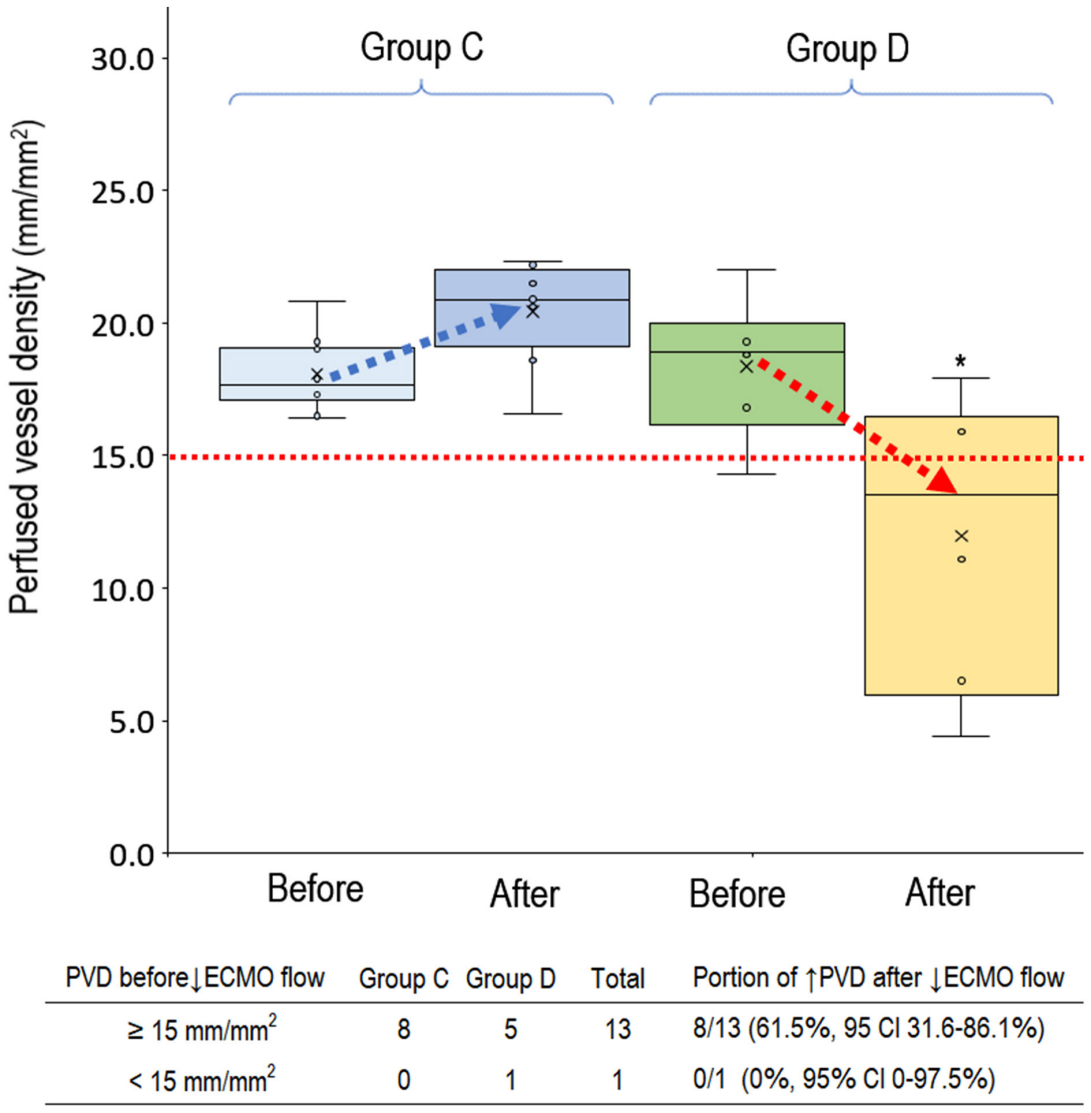
Perfused small vessel before and after decreasing venoarterial extracorporeal membrane oxygenation pump flow. Group C (*n* = 8), PVD increased after decreasing VA-ECMO pump flow; group D (*n* = 6), PVD decreased after decreasing VA-ECMO pump flow. PVD before decreasing ECMO pump flow did not differ significantly between group C and D (*P* = 0.698). PVD was higher in the group C than in group D after decreasing ECMO pump flow (*P* = 0.003). ^*^P < 0.05 comparison between group C and D. CI, confidence interval; PVD, perfused vessel density; VA-ECMO, venoarterial extracorporeal membrane oxygenation.

**Figure 6:**
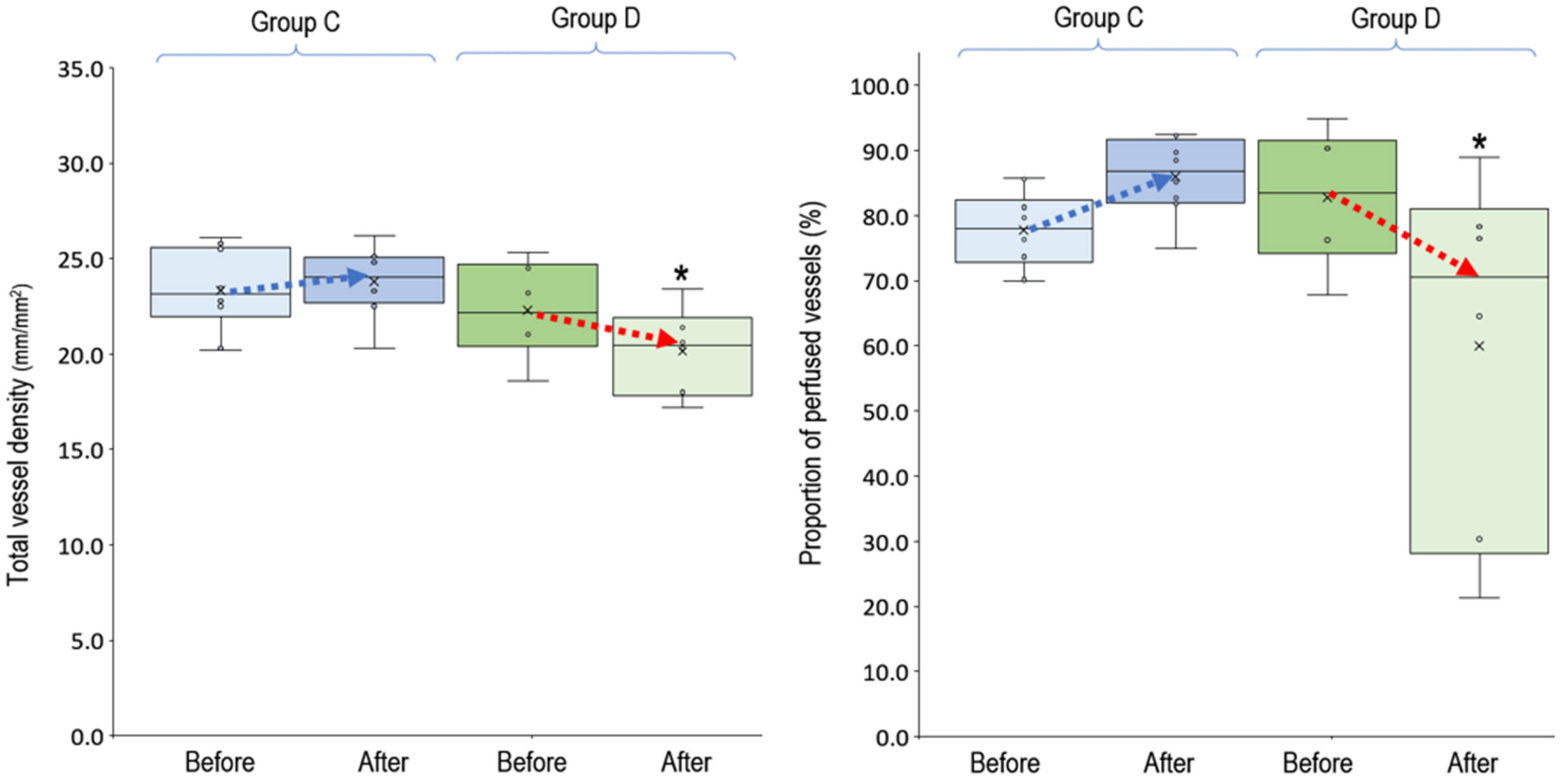
Total vessel density and proportion of perfused vessels before and after decreasing venoarterial extracorporeal membrane oxygen pump flow. Group C (*n* = 8), PVD increased after decreasing VA-ECMO pump flow; group D (*n* = 6), PVD decreased after decreasing VA-ECMO pump flow. ^*^P < 0.05 comparison between group C and D using independent-samples median tests. *n*, number of events; PVD, perfused vessel density; PPV, proportion of perfused vessels; TVD, total vessel density; VA-ECMO, venoarterial extracorporeal membrane oxygenation.

In the 14 clinical events of decreasing VA-ECMO pump flow, we observed 6 (42.9%, 95% CI, 17.7–71.1%) events with decreased PVD (Figure 5). TVD and PPV before and after decreasing VA-ECMO pump flow are presented in Figure 6. PVD, TVD, and PPV before decreasing VA-ECMO pump flow were not significantly correlated to their changes after decreasing VA-ECMO pump flow. In *post-hoc* analysis, PVD, VA-ECMO pump flow, MAP, lactate level, and inotropic score before decreasing the VA-ECOM pump flow did not differ significantly between group C and D.

## Discussion

This study revealed both contradictory and non-contradictory responses of microcirculation to changes in VA-ECMO pump flow. This finding suggested that adjusting VA-ECMO pump flow according to current treatment goals might not ensure a good microcirculation. Moreover, we found that PVD < 15 mm/mm^2^ before increasing VA-ECMO pump flow is associated with a higher probability to increase PVD after increasing VA-ECMO pump flow.

The finding that PVD < 15 mm/mm^2^ at baseline had better response to increasing VA-ECMO pump flow is compatible with several previous studies of microcirculation resuscitation. First, red blood cell transfusion improves microcirculation in those patients with impaired microvascular flow at baseline (25–27). Second, fluid therapy improve microvascular flow in patients with abnormal microvascular flow at baseline, but not in patients with normal microvascular flow at baseline (28). Third, dobutamine only improved sublingual microcirculation in patients with severe alteration at baseline (29). These studies had a common finding that dissociation between microcirculation and systemic hemodynamics was frequently seen. It suggests that only direct measurement of the microcirculation before and after the treatments can see the real response of microcirculation (30).

The finding of contradictory decrease in PVD after increasing VA-EMCO pump flow is compatible with that of Busch et al. (31). They suggest that cerebral blood flow and oxygenation are not well-predicted by VA-ECMO pump flow or blood pressure. Moreover, increasing VA-ECMO pump flow may have two effects on patient's own cardiac output. First, increased venous drainage of VA-ECMO decrease venous return of blood flow to the right atrium. Second, increased VA-ECMO arterial blood flow increases cardiac afterload (32, 33). The decreased preload and increased afterload may reduce the patient's own cardiac output. In patients with VA-ECMO, head and brain perfusions are determined according to the balance between VA-ECMO pump flow and the patient's own cardiac output (34, 35). Further studies are required to investigate whether sublingual microcirculation is correlated with cerebral blood flow in patients with VA-ECMO.

Timely weaning of VA-ECMO can reduce its associated complications and shorten its duration. The finding of decrease in PVD after decreasing VA-ECMO pump flow is compatible with the study of Akin et al. (11). They suggest that sublingual microcirculation is a novel potential marker for identifying successful weaning from VA-ECMO. There are two differences between these two studies. First, we investigated the microcirculatory response to decreasing VA-ECMO pump flow within 48 h after placement of VA-ECMO, and Akin et al. investigated the microcirculatory response to weaning VA-ECMO pump flow at 48 h or up to 3 weeks after placement of VA-ECMO. Second, the decreases in VA-ECMO pump flow were 10 to 30% of the baseline value in our study, and the decreases in VA-ECMO pump flow were 50% of the baseline value in the study of Akin et al. (11). Therefore, our study further identified that sublingual microcirculation might be a potential marker for ensuring adequate tissue perfusion during early weaning VA-ECMO pump flow.

This study has several limitations. First, all patients received peripheral VA-ECMO, and the microcirculatory response to changes in VA-ECMO pump flow might be different in those patients with central VA-ECMO. Second, this observational pilot study was not powered to find out the predictors of microcirculation response after changing VA-ECMO pump flow. However, our preliminary results show that lower PVD before increasing VA-ECMO pump was associated with increased PVD after increasing VA-ECMO flow. Other parameters before increasing VA-ECMO pump flow, include ScvO_2_ and inotropic score, are warranted for further investigation. Third, this study enrolled patients with different indications of VA-ECMO support. Further studies with specific indication of VA-ECMO support are warranted to investigate the predictors for contradictory responses of microcirculation after changing the VA-ECMO pump flow. Fourth, further studies with more enrolled patients and more time points of microcirculation examinations are warranted to investigate whether aiming to maintain an adequate microcirculation after changing VA-ECMO pump flow can ensure better clinical outcomes.

## Conclusion

Our study revealed both contradictory and non-contradictory responses of sublingual microcirculation to changing VA-ECMO pump flow. At this stage, tandem measurements of microcirculation before and after changing VA-ECMO flow may help to ensure a good microcirculation.

## Data Availability Statement

The datasets presented in this article are not readily available because of the regulation of the Research Ethics Committee of authors' hospital. The datasets used and/or analyzed during the current study are available from the corresponding author on reasonable request. Requests to access the datasets should be directed to Yu-Chang Yeh, tonyyeh@ntuh.gov.tw.

## Ethics Statement

The studies involving human participants were reviewed and approved by Research Ethics Committee of National Taiwan University Hospital. The patients/participants provided their written informed consent to participate in this study.

## Author Contributions

T-JW, C-HW, W-SC, CI, T-YL, and Y-CY: concept and design. T-JW, C-HW, C-HH, C-HL, and Y-CY: patient enrollment and data collection. T-JW, W-SC, CI, T-YL, and Y-CY: interpretation of data. T-JW, W-SC, and Y-CY: drafting manuscript. C-HW, C-HH, M-JW, Y-SC, CI, T-YL, and Y-CY: critical revision of the manuscript. M-JW and Y-SC: study supervision. All authors: contributed to the article and approved the submitted version.

## Conflict of Interest

The authors declare that the research was conducted in the absence of any commercial or financial relationships that could be construed as a potential conflict of interest.
